# The staphylococcal biofilm protein Aap mediates cell–cell adhesion through mechanically distinct homophilic and lectin interactions

**DOI:** 10.1093/pnasnexus/pgac278

**Published:** 2022-12-02

**Authors:** Can Wang, Constance Chantraine, Albertus Viljoen, Andrew B Herr, Paul D Fey, Alexander R Horswill, Marion Mathelié-Guinlet, Yves F Dufrêne

**Affiliations:** Louvain Institute of Biomolecular Science and Technology, UCLouvain, Croix du Sud, 4-5, bte L7.07.07, B-1348 Louvain-la-Neuve, Belgium; Louvain Institute of Biomolecular Science and Technology, UCLouvain, Croix du Sud, 4-5, bte L7.07.07, B-1348 Louvain-la-Neuve, Belgium; Louvain Institute of Biomolecular Science and Technology, UCLouvain, Croix du Sud, 4-5, bte L7.07.07, B-1348 Louvain-la-Neuve, Belgium; Divisions of Immunobiology and Infectious Diseases, Cincinnati Children’s Hospital Medical Center, Cincinnati, OH 45229, USA; Department of Pathology and Microbiology, University of Nebraska Medical Center, Omaha, NE 68198, USA; Department of Immunology and Microbiology, University of Colorado School of Medicine, Aurora, CO 80045, USA; Louvain Institute of Biomolecular Science and Technology, UCLouvain, Croix du Sud, 4-5, bte L7.07.07, B-1348 Louvain-la-Neuve, Belgium; Louvain Institute of Biomolecular Science and Technology, UCLouvain, Croix du Sud, 4-5, bte L7.07.07, B-1348 Louvain-la-Neuve, Belgium

**Keywords:** Aap, intercellular adhesion, lectin binding, homophilic interaction, mechanical strength

## Abstract

The accumulation phase of staphylococcal biofilms relies on both the production of an extracellular polysaccharide matrix and the expression of bacterial surface proteins. A prototypical example of such adhesive proteins is the long multidomain protein Aap (accumulation-associated protein) from *Staphylococcus epidermidis*, which mediates zinc-dependent homophilic interactions between Aap B-repeat regions through molecular forces that have not been investigated yet. Here, we unravel the remarkable mechanical strength of single Aap–Aap homophilic bonds between living bacteria and we demonstrate that intercellular adhesion also involves sugar binding through the lectin domain of the Aap A region. We find that the mechanical force needed to unfold individual β-sheet-rich G5-E domains from the Aap B-repeat regions is very high, ranging from 300 up to 1,000 pN at high loading rates, indicating these are extremely stable. This high mechanostability provides a means to the cells to form highly adhesive and cohesive biofilms capable of sustaining high physiological shear stress. Importantly, we identify a previously undescribed role of Aap in bacterial–bacterial adhesion, that is, heterophilic sugar binding by a specific lectin domain located in the N-terminal A region, which might be important to establish initial contacts between cells before strong homophilic bonds come into play. This study emphasizes the remarkable mechanical and binding properties of Aap as well as its wide diversity of adhesive functions.

Significance Statement The pathogenicity of staphylococci is associated with the ability of the bacteria to attach to indwelling devices and host tissues and to favor cell–cell adhesion, leading to the formation of highly cohesive biofilms. The *Staphylococcus epidermidis* multidomain protein Aap promotes zinc-dependent homophilic interactions between Aap B-repeat regions of neighboring bacteria, but the forces and dynamics of self-association are currently unknown. By means of single-molecule experiments combined with genetic manipulation, we unravel the mechanical strength of single Aap–Aap homophilic bonds. Strikingly, we also identify and dissect a novel mechanism by which Aap mediates intercellular adhesion through heterophilic sugar binding by its lectin A domain. Our results offer promise for the development of antiadhesive therapeutics targeting cell–cell interactions and biofilm formation.

## Introduction

*Staphylococcus epidermidis* is a commensal of the human skin able to turn into an opportunistic pathogen (1), causing biomaterial-associated infections, which commonly leads to bloodstream infections (2, 3). Pathogenicity and chronic persistence of this pathogen are associated with its ability to attach to the surface of indwelling devices, such as catheters and prostheses, using a repertoire of cell wall-anchored (CWA) proteins (4–7). Following this initial adhesion step, the cells aggregate during the accumulation phase, leading to microcolonies and to the formation of highly adhesive and cohesive biofilms (8, 9). Importantly, *S.epidermidis* is the leading cause of device-related infections, and its ability to form stable biofilms eventually triggers and exacerbates the severity of specific skin diseases such as atopic dermatitis (10–12), highlighting the importance of understanding the molecular details of bacterial–bacterial interactions and their implications in biofilm maturation and subsequent device-related infections.

**Fig. 1.**
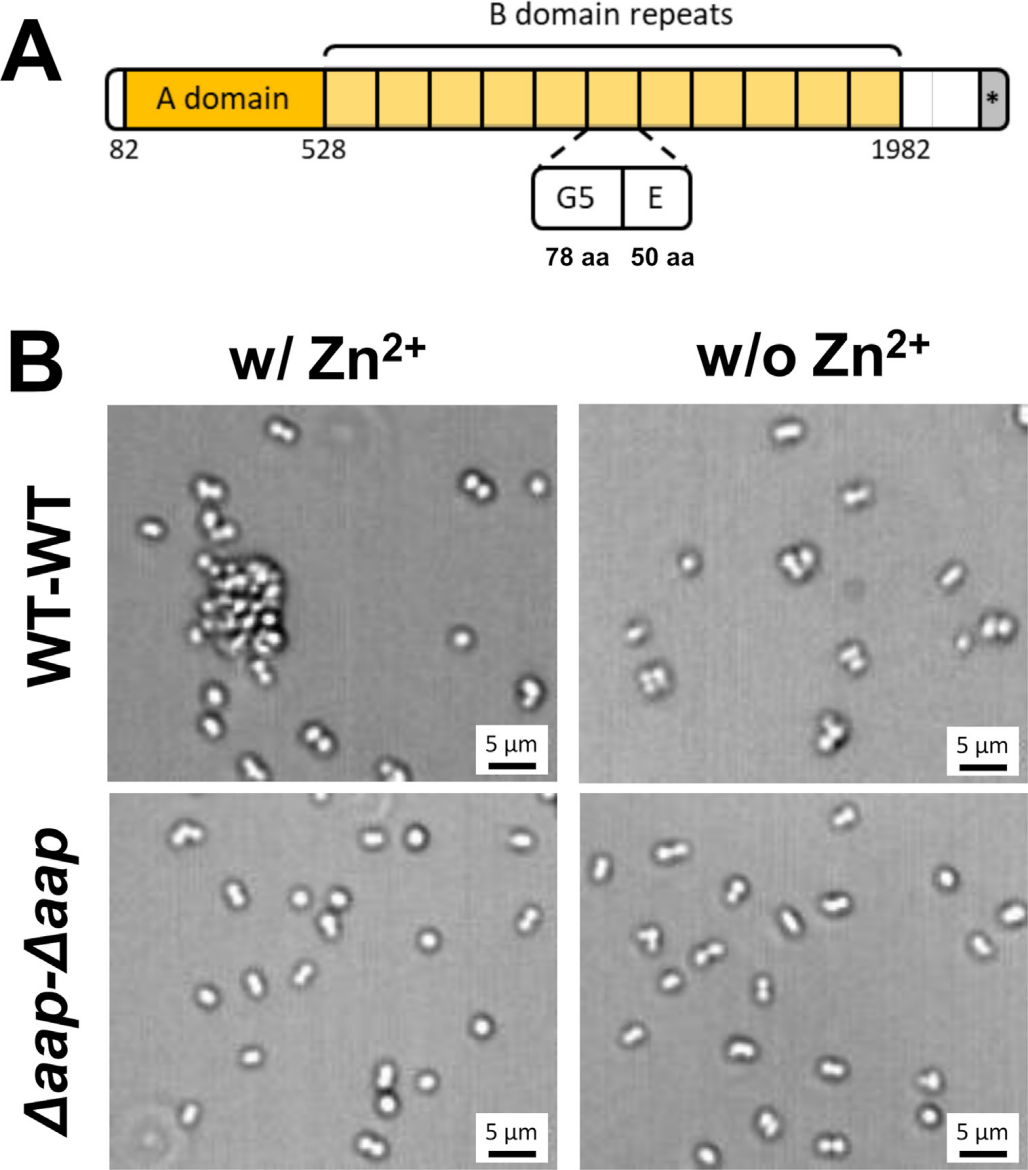
Zinc-dependent role of Aap in cell–cell adhesion. (A) Schematic representation of Aap expressed by *S.epidermidis* CSF41498 strain. Aap consists of an A region (including a lectin-like domain and a variable number of 16 aa repeats, dark yellow), a B-repeat region (light yellow) containing 11 tandem E-G5 domains (50 and 78 aa, respectively), a proline/glycine-rich region, and a cell wall anchoring motif (*) (LPDTG). (B) Optical microscopy images of *S.epidermidis* cells expressing full-length Aap (WT cells) or *S.epidermidis* expressing no Aap (*Δaap* cells) after resuspension in TBS buffer with or without 1 mM Zn^2+^.

Traditionally, staphylococcal cell–cell adhesion has been considered to be driven by electrostatic interactions involving the positively charged polysaccharide intercellular adhesin (PIA), also known as the poly-*N*-acetylglucosamine (PNAG) (13–15). However, various CWA proteins also critically support intercellular adhesion (6, 16–20). An archetype in *S.epidermidis* is the accumulation-associated protein (Aap) (7, 17), an ortholog of the *S. aureus* protein SasG (21), which mediates cell–cell association through zinc-dependent homophilic bonds between Aap molecules on opposing cells (20). This elongated protein contains an N-terminal A domain composed of a A-repeat region (11 partially conserved 16-residue repeats) and a 222-amino acid l-type lectin domain, followed by a B-repeat region consisting of up to 17 nearly identical sequence repeats of 128-amino acid sequences (20, 22). B repeats are made of G5 domains (78 residues, see cartoon) in a tandem array, separated by E regions of 50 residues (Fig. 1A). Homophilic interactions involved in biofilm formation result from the modular assembly of individual G5-E domains along the length of B repeats on adjacent cells (20, 22). The current model is that the two interacting Aap B-regions form a rope-like antiparallel twisted structure (22). Interestingly, B repeats exhibit a monomer–dimer–tetramer reversible equilibrium in the presence of zinc, resulting in the formation of functional amyloid fibers within the biofilm (23). The Aap A region is not engaged in such homophilic interactions but is involved in specific binding to host surfaces (24). Specifically, recent studies have found that the lectin domain within the A region is essential for adherence of *S.epidermidis* to host glycans in the stratum corneum in healthy human skin (25).

Two crucial, yet unsolved issues associated with the biofilm formation and accumulation phases are as follows: (i) what is the mechanical strength of Aap homophilic bonds and (ii) are there other mechanisms than homophilic binding that could play a role in cell–cell adhesion. Here, we sought to answer these questions using single-molecule experiments on living bacteria (26, 27), hence using Aap proteins in their fully physiological and functional states. We used the strain *S.epidermidis* CSF41498 that exhibits minimal auto-processing of Aap, thus producing mostly full-length Aap molecules with both the A and B regions but also, to a lower extent, cleaved Aap only showing the B region (28). This is opposed to other strains like *S.epidermidis* 1457 showing high processing and A region cleavage of Aap (29). The results show that the strength of single homophilic bonds is very high (forces up to 1,000 pN), because of the high mechanical stability of the G5-E domains of the B region, explaining how the pathogen can form highly adhesive and cohesive biofilms capable of withstanding high shear stress. In addition, a novel type of interaction is identified, that is, heterophilic sugar binding by the A region lectin domain, which could favor initial cell–cell contacts before stronger homophilic interactions take place. By identifying the critical structural determinants of *S.epidermidis* co-adhesion, this study shows promise for the future design of new inhibitors (peptides, sugars, and antibodies) capable of preventing cell–cell adhesion and biofilm growth.

## Results

### Aap mediates intercellular adhesion

Previous investigations (17–20, 22) have demonstrated the key role of the Aap B-region in driving *S. epidermidis* cell–cell adhesion and biofilm formation. To confirm this role in our working conditions, we checked the ability of *S.epidermidis* CSF41498 (hereafter WT) to co-aggregate at the microscale (Fig. 1B). While in the absence of Zn^2+^ the WT strain expressing Aap mostly appeared as isolated or paired cells randomly distributed on the substrate, addition of 1 mM Zn^2+^ for 15 min led to the formation of cell clusters up to 10 μm in size. As expected, the CSF41498 *Δaap* mutant, lacking the Aap adhesins (hereafter *Δaap*), did not form any large aggregates, even in the presence of Zn^2+^, consistent with the absence of cell–cell adhesion. Paired cells that were sometimes observed in all conditions are very likely to reflect two dividing cells.

### Strength of single Aap homophilic bonds

**Fig. 2.**
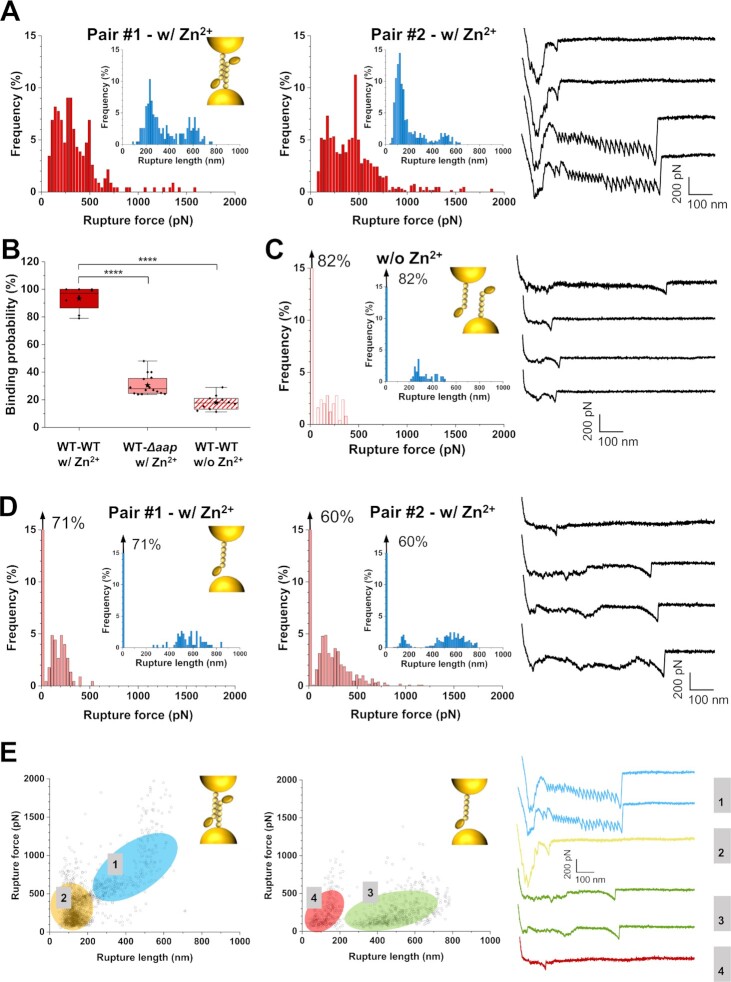
Aap mediates intercellular adhesion. Rupture force and rupture length histograms (inset) of two representative WT–WT cell pairs in the presence of zinc (A*, n* = 256 curves for each histogram), one representative WT–WT cell pair in the absence of zinc (C, *n* = 256 curves), and two representative WT–*Δaap* cell pairs in the presence of zinc (D, *n* = 256 curves for each histogram) obtained by SCFS. Corresponding schematic representations of the interactions (inset) and representative retraction force profiles (right) are presented for all conditions. (B) Box plots comparing the adhesion probability of WT–WT cell pairs with (*n* = 8 pairs) or without (*n* = 10) zinc, and WT–*Δaap* cell pairs with zinc (*n* = 16). Stars are the mean values, lines the medians, boxes the 25–75% quartiles, and whiskers the SD. ^****^P < 0.0001. (E) Plots of rupture force as a function of rupture length displaying different interaction signatures for WT–WT (left) and WT–*Δaap* (middle) pairs in the presence of zinc. Representative retraction force profiles are presented for each type of population. If present, the arrow at the top left of a histogram stands for the nonadhesive events. All force curves were obtained with an applied force of 250 pN, and a retraction velocity of 1 µm s^−1^. See more cell pairs in the Appendix in Supplementary Material, Fig. S1.

We then investigated the strength of homophilic bonds between two living bacteria, by using single-cell force spectroscopy (SCFS; Fig. 2). A single cell was attached to an AFM probe, enabling us to record force–distance (FD) curves toward another isolated cell immobilized on a substrate. Fig. 2A presents the rupture forces and rupture lengths determined from those FD curves, for two representative pairs of WT cells in the presence of zinc (for more pairs, see Appendix in Supplementary Material, Fig. S1A). The distribution of forces featured two maxima (Fig. 2E): a first one centered at 291 ± 171 pN (mean ± SD from *n* = 1,829 curves from eight independent cell pairs) and a second at 592 ± 133 pN (*n* = 509 curves). These two types of events ruptured at different distances, 135 ± 30 and 374 ± 115 nm for the low and high force events, respectively. The two populations strongly differed in their force profiles, in that weak adhesive events displayed sharp single peaks while strong ones showed sawtooth patterns with multiple equally spaced peaks. In the absence of zinc, the binding probability dramatically dropped from 93 to 18%, with adhesive events of 214 ± 90 pN breaking at 402 ± 136 nm (*n* = 426 curves from 10 independent cell pairs, Figs. 2B and C; for more pairs, see Appendix in Supplementary Material, Fig. S1B). Moreover, sawtooth unfolding patterns were not observed anymore. This demonstrates the key role of zinc-dependent Aap dimers in co-adhesion, and suggests that the very short ranged peaks also reflect zinc-dependent Aap associations, perhaps between larger multimers or preformed amyloids occurring on the cell surface (23).

To test whether the above forces originate solely from homophilic Aap–Aap association, we performed similar SCFS experiments between WT and *Δaap* bacteria, under the same conditions, that is, in the presence of zinc. The adhesion substantially decreased but was still unexpectedly high, ∼30% (*n* = 16 pairs, Fig. 2B). Adhesive events mainly showed single peaks of 187 ± 55 pN that ruptured at 512 ± 67 nm (Figs. 2D and E; for more pairs, see Appendix in Supplementary Material, Fig. S1C), thus significantly different from the WT–WT adhesion signatures. Because multiunfolding signatures were never observed in WT–*Δaap* cells co-adhesion (Fig. 2D), we conclude that such signatures solely reflect homophilic binding between Aap rather than ligand-binding. These homophilic interactions arise from the self-assembly of the B repeats, which subsequently unfold and rupture under high external tensile loading. On the other hand, adhesive signatures reported between WT–*∆aap* cells suggest that heterophilic interactions are at play between Aap, and an unidentified ligand on the partner mutant cell. Interestingly, this behavior was relatively similar to the one of WT–WT pairs without zinc, suggesting it might involve similar, and novel, nonzinc dependent heterophilic interactions. The lower binding probability observed for WT–WT pairs in the absence of zinc may be due to steric issues arising from the dense expression of Aap molecules on both WT cells opposed to the *Δ*aap cells, which likely exposing more ligands at their surface. Altogether, these results indicate (i) that the Aap–Aap homophilic bonds formed by the trans assembly of B-domains (20, 22) are mechanically strong, and (ii) that the protein is also engaged in a previously unidentified heterophilic ligand interaction.

### The high mechanostability of the Aap B-domain

**Fig. 3.**
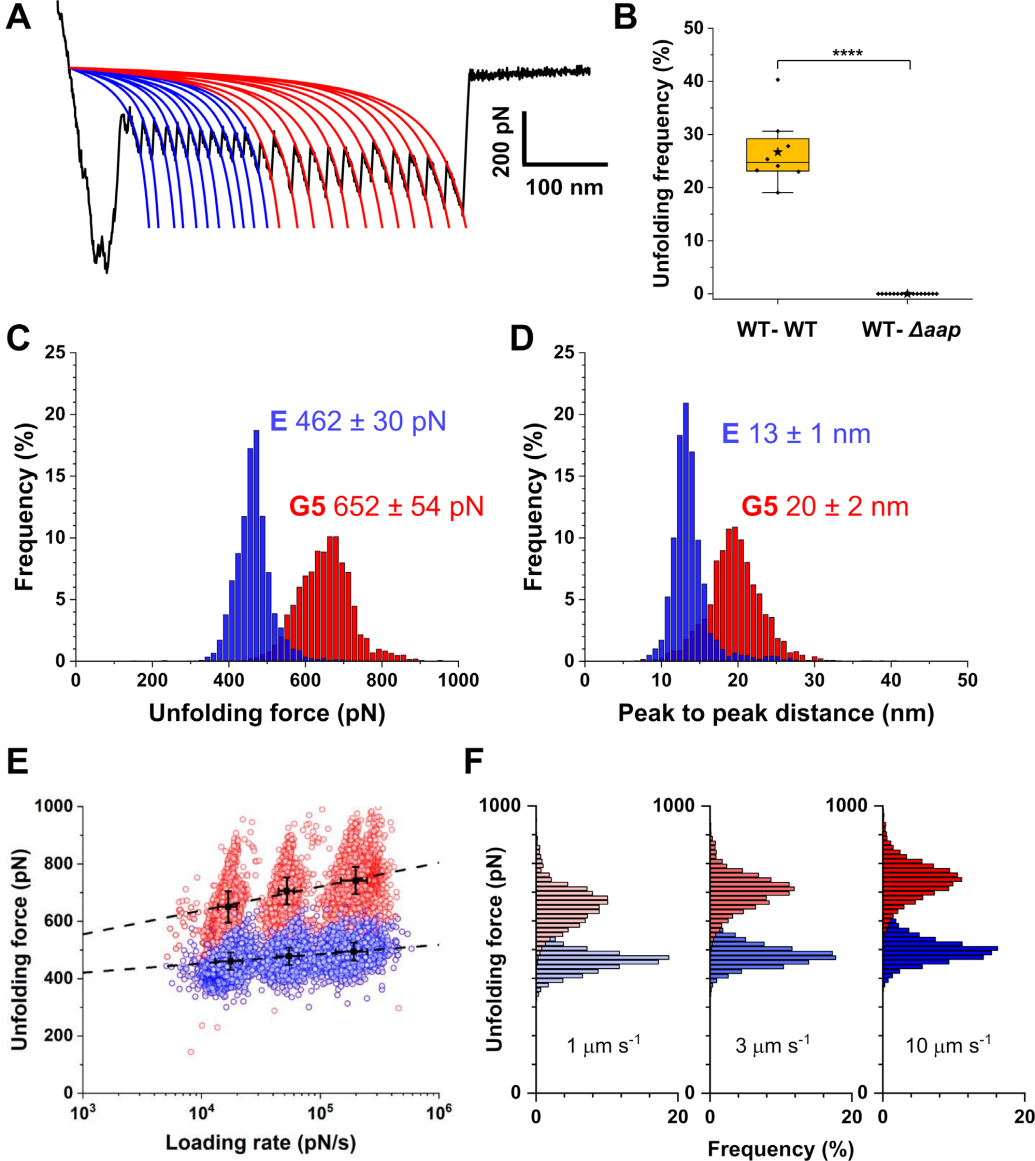
Mechanostability of the Aap B-domain is high and activated by tensile loading. (A) Representative force curve displaying typical sawtooth profile well fitted with the WLC model (blue and red lines). Up to 11 low force peaks followed by 11 high force peaks are observed, reflecting the unfolding of E and G5 repeats, respectively. (B) Box plot showing the unfolding frequency observed for WT–WT cell pairs (*n* = 8 pairs) and WT–*∆aap* cell pairs (*n* = 16 pairs) in the presence of zinc. Stars are the mean values, lines the medians, boxes the 25–75% quartiles and whiskers the SD. ^****^P < 0.0001. (C, D) Histograms of unfolding forces (C) and peak-to-peak distances (D) obtained by analyzing multiple unfolding patterns of WT–WT pairs (*n* = 3548 peaks for E in blue; *n* = 2743 peaks for G5 in red, from three independent cell pairs). (E) Dynamic force spectrum of the unfolding of single Aap E (in blue) and G5 (in red) subdomains (*n* = ∼18,406 unfolding peaks in total from 3 representative WT–WT pairs) at various retraction velocities (1, 3, and 10 μm s^−1^). The black dotted line stands for the Bell–Evans (BE) fit from which the energetic barrier and off-rate constant are extracted: G5, *x_u_* = 0.1 nm and *k*_off_^0^ = 6.1 × 10^−6^ s^−1^; E, *x_u_* = 0.3 nm, and *k*_off_^0^ = 7.1 × 10^−12^ s^−1^. (F) Distribution of E (in blue) and G5 (in red) unfolding forces at the different retraction velocities, further illustrating the increase in force with the retraction velocity.

To get further insight into this high stability, we further dissected the sawtooth patterns (Fig. 3A). These sequential force peaks, which account for ∼27% of all reported adhesive events in WT pairs (Fig. 3B), are reminiscent of those observed when stretching modular proteins like titin (30), and typically result from the force-induced unfolding of secondary structures. Most sawtooth profiles featured two distinct force peaks, a first group of ∼11 sequential, equally spaced, force peaks (462 ± 30 pN, *n* > 2,700 peaks from three independent cell pairs), always followed by another group ∼11 of high force peaks (652 ± 54 pN) (Fig. 3C). All those peaks were well fitted by the worm-like chain (WLC) model, as expected for protein domains unfolding (Fig. 3A). The peak-to-peak distances were centered at 13 ± 1 and 20 ± 2 nm for the low and high force peaks, respectively (Fig. 3D). Assuming that each residue contributes 0.36 nm to the contour length of a fully extended polypeptide chain and that the folded lengths of the E and G5 repeats of the B region of Aap are 4.5 and 7.0 nm (31), the measured peak inter-distances match well with the unfolding of the E and G5 domains (50 and 78 residues, respectively).

Dynamic force spectroscopy (DFS) plots were then obtained for the WT–WT E and G5 unfolding behavior, by varying the pulling speed and in turn the loading rate (LR, estimated from the force vs time curves). As predicted by the Bell-Evans (BE) theory, both unfolding forces of E and G5 repeats increased linearly with the logarithm of the LR (Fig. 3E and F). From this model, the position of the energy barrier that separates the bound from the unbound state was extracted, *x*_u_ = 0.3 nm and 0.1 nm for E and G5, and off-rate constants at thermal equilibrium were determined, *k*_off_^0^ = 7.1 × 10^−12^ s^−1^ and *k*_off_^0^ = 6.1 × 10^−6^ s^−1^. These results illustrate the high mechanostability of the Aap B-repeats self-association.

### The Aap A-domain engages in lectin-sugar cell–cell interactions

**Fig. 4.**
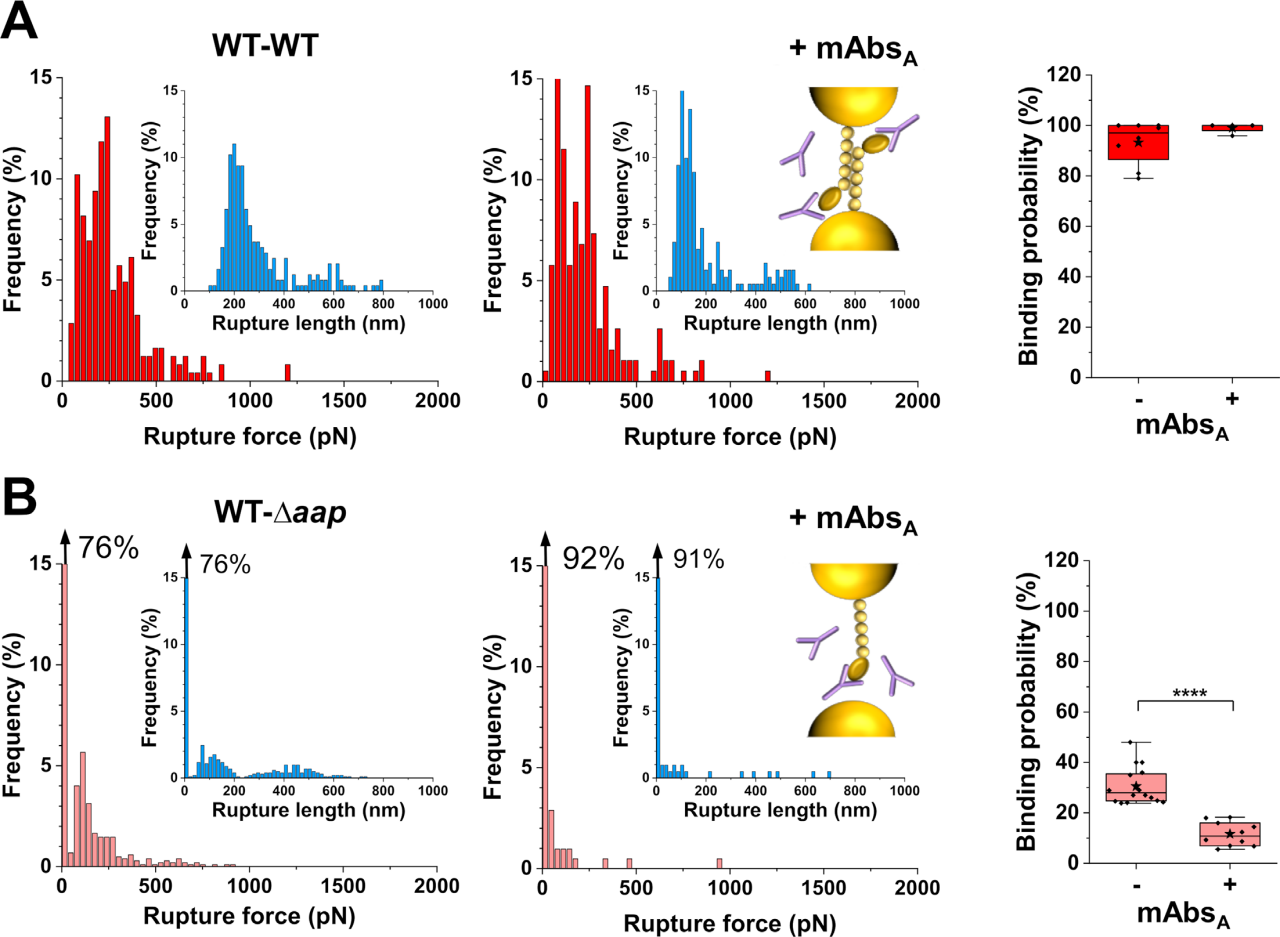
The Aap A-domain engages in cell–cell interactions. (A) Rupture force and rupture length (inset) histograms for one representative WT–WT cell pair before and after treatment with 1 mM mAbs directed against the A domain of Aap (mAbs_A_). (B) Same data for one representative WT–*Δaap* cell pair. At the right, box plots of the binding probability in presence or absence of mAbs_A_ highlighting their absence of effect on WT–WT pairs (*n* = 8 pairs and *n* = 4 pairs, respectively) and their inhibition effect on WT–*∆aap* cell pairs (*n* = 16 and *n* = 10 pairs, respectively). Stars are the mean values, lines the medians, boxes the 25–75% quartiles and whiskers the SD. ^****^P < 0.0001. If present, the arrow at the top left of a histogram stands for the nonadhesive events. All force curves were obtained with an applied force of 250 pN, and a retraction velocity of 1 µm s^−1^. See more cell pairs in the Appendix in Supplementary Material, Fig. S2.

While we fully characterized the mechanical strength of Aap homophilic interactions, the question remains on how the protein might also engage in heterophilic interactions during cell–cell association. To answer this, we measured the forces between WT–*Δaap* pairs, following treatment with monoclonal antibodies (mAbs) directed against the A region of Aap (mAbs_A_) (Fig. 4 and Appendix in Supplementary Material, Fig. S2) (32–34). Whereas rupture forces and binding probability were not altered for the WT–WT pairs (Fig. 4A, Appendix in Supplementary Material, Fig. S2A), the co-adhesion between WT and *Δaap* cells significantly dropped (Fig. 4B, Appendix in Supplementary Material, Fig. S2B). This leads us to conclude that homophilic bonds only require the B region of Aap, and that, unexpectedly, the A region engages into some type of heterophilic interaction with the surface of *Δaap* cells.

**Fig. 5.**
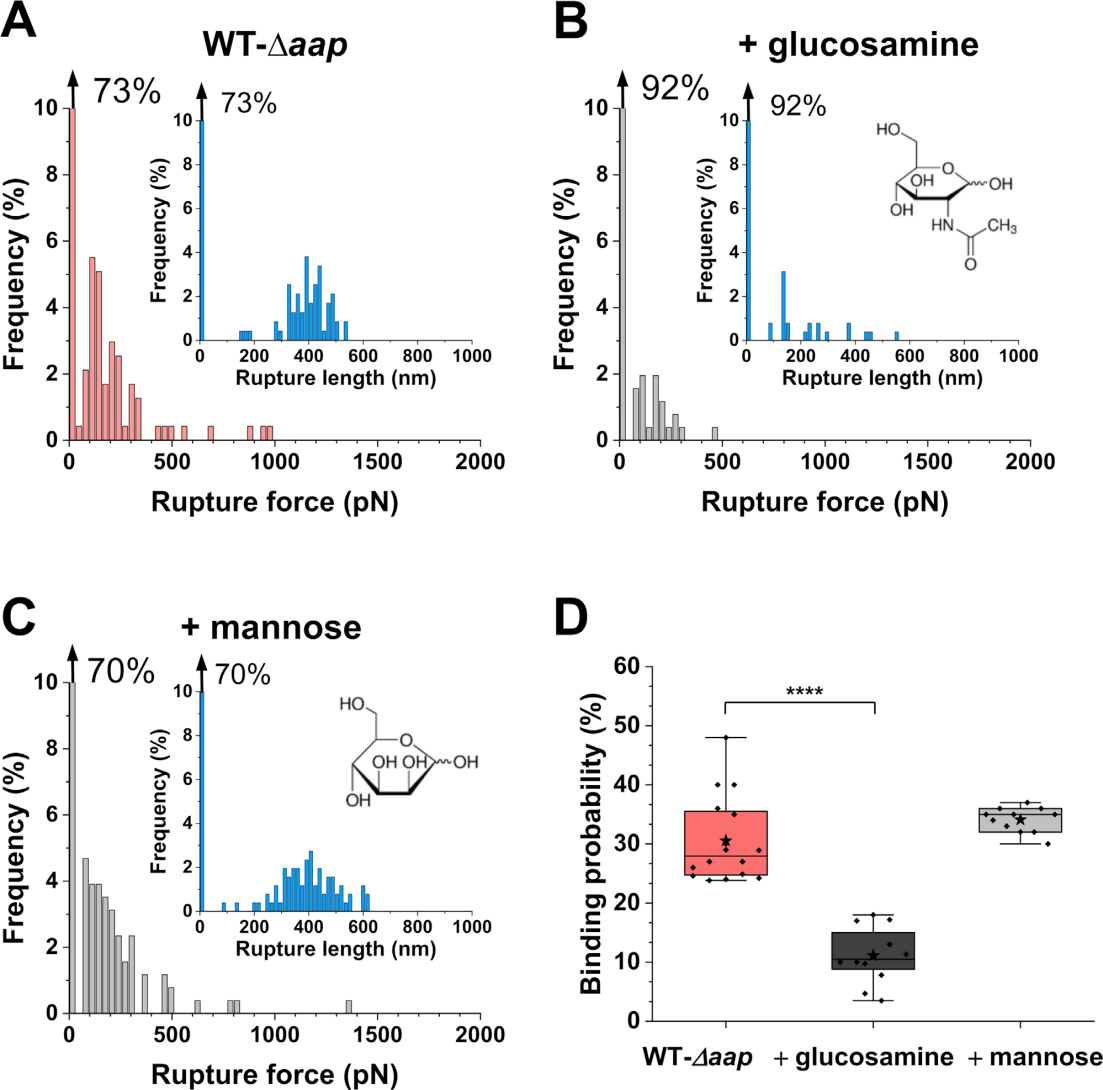
Cell aggregation involves lectin-sugar binding. Rupture force and rupture length (inset) histograms for one representative WT–*Δaap* pair before (A) and after the addition of 1 mM *N*-acetyl-d-glucosamine (B).(C) Histograms for a representative WT–*Δaap* pair treated with 1 mM mannose. (D) Box plot comparing the binding probability of WT–*Δaap* pairs in classical conditions (*n* = 16 pairs), treated with 1 mM *N*-acetyl-d-glucosamine (*n* = 12 pairs), or with 1 mM mannose (*n* = 11 pairs). Stars are the mean values, lines the medians, boxes the 25–75% quartiles and whiskers the SD. ^****^P < 0.0001. If present, the arrow at the top left of a histogram stands for the nonadhesive events. More cell pairs are presented in the Appendix in Supplementary Material, Fig. S3.

We then wonder what is the molecular nature of this heterophilic interaction. The Aap lectin domain within the A region has been shown to bind human nasal epithelial cells (35) and human corneocytes (24, 25). Given the shape of the WT–*Δaap* force peaks, we postulated they may reflect the binding of specific carbohydrates on one cell to the A lectin domain on another cell. Recently, by means of mutagenesis and binding assays to corneocytes, it has been shown that *N*-acetylglucosamine, a glycan found on the bacterial surface, might serve as a potential binding partner for the Aap lectin domain of *S.epidermidis* 1457 *Δica* (25). Thus, to assess if the lectin domain might be involved in the observed heterophilic interactions, we performed blocking experiments, injecting such known glycan ligand, *N*-acetyl-d-glucosamine, at a final concentration of 1 mM (Fig. 5 and Appendix in Supplementary Material, Fig. S3). The adhesion frequency between WT and *Δaap* cells significantly dropped from 30 to 11% after injection of *N*-acetyl-d-glucosamine (Fig. 5D). The remaining adhesive events were similar to those observed before injection (Fig. 5A and B and Appendix in Supplementary Material, Fig. S3A), suggesting that the same single interaction was probed but not fully blocked, as often observed in AFM experiments. The blocking activity was not observed when using mannose, suggesting that lectin binding is specific (Fig. 5C and D and Appendix in Supplementary Material, Fig. S3B).

Remarkably, interactions between WT pairs in the absence of zinc, which showed similar adhesion signatures as WT–*Δaap* pairs, were also inhibited by *N*-acetyl-d-glucosamine with a significant decrease of binding probability from 18 to 8% (Appendix in Supplementary Material, Fig. S4). Collectively, these results support (i) the essential and distinct roles of Aap in cell–cell adhesion via its A and B regions, and (ii) the existence of novel heterophilic interactions between the lectin domain of the Aap A region and *N*-acteyl-d-glucosamine, a common sugar found on bacterial surfaces.

## Discussion

Biofilm formation on indwelling medical devices and host cells is promoted by extracellular polysaccharides and CWA proteins, which both mediate intercellular adhesions. *Staphylococcusepidermidis* expresses several CWA surface proteins involved in adherence to host substrates, biofilm growth and stability, and in subsequent infections (7). The multidomain protein Aap promotes zinc-dependent homophilic interactions between Aap B-repeat regions of neighboring bacteria, but the molecular forces and dynamics of self-association involved are currently unknown. Here, we have unraveled the strength, specificity, and dynamics of *S.epidermidis* homophilic bonds, and have identified and dissected a novel mechanism by which Aap mediates intercellular adhesion, that is, through heterophilic sugar binding by the lectin domain of the A region.

The force needed to unfold G5-E repeats in the B domains and to rupture single homophilic bonds are much larger than the unfolding forces of most β-fold multidomain proteins, typically in the 100 to 250 pN range (36). However, the high Aap forces well agree with MD simulations and in vitro experiments on the homologous *S.aureus* SasG protein (31). It was shown that the high mechanostability of the B repeats originates from tandemly arrayed mechanical clamps involving long stretches of hydrogen bonds and associated side-chain packing interactions along the β-strands. In addition, the observed mechanostability of the Aap B repeats is consistent with the strong stabilization the Aap repeating G5-E-G5-E pattern provides to the overall fold through nearest-neighbor interactions between domains. Indeed, it has been shown that removal of the hydrophobic stacking interaction in the interface between the E and G5 domains resulted in the unfolding of the G5 domain downstream of the interface (22). Likewise, the B-repeat region of *S.aureus* ortholog SasG was shown to exhibit cooperative folding, with interfaces between domains that contribute more stability than the G5 and E domains provide individually (31). Further, the presence of only one G5-E domain in SasG was shown to mediate much lower forces in homophilic interactions than the entire B-repeat region, highlighting the key role of repeat-multiplication in sustaining high mechanostability (37). Consequently, force-induced unfolding of a single domain in one Aap molecule would destabilize the entire B-repeat region due to the loss of the interface stabilization, leading to the observed unfolding pattern and rupture lengths.

Under external mechanical forces, the E and G5 domains will unfold sequentially, acting as force buffers capable of relieving mechanical stress. Under tension, the protein may become softer, than in its stiff folded state when no force is applied. The high mechanical strength of the Aap–Aap bond is of biological relevance as Aap has been shown to be critical for biofilm formation under fluid shear conditions (38). We expect that strong Aap homophilic bonds will play an essential role in favoring tight cell–cell contacts and stable colonies.

What is the structural basis for the observed homophilic force profiles? X-ray crystallography analysis (22) has suggested a twisted rope-like structure between bacterial cells, in which the antiparallel monomers wrap around one another. The β-sheet unfoldings we observed when separating two cells are therefore very likely to result from the rupture of rope-like bonds. Our unfolding patterns and rupture lengths match those of a single Aap and SasG (31) adhesins, indicating that the proteins are interacting in trans, and that most likely only a single adhesin unfolds during the force-induced bond rupture. Unfolding of two Aap would require similar pulling geometries as well as the same protein conformation and orientation, which is unlikely to occur under our in vivo physiologically relevant conditions. In addition, simultaneous unfolding would lead to higher forces and rupture lengths, which was never observed. We therefore believe that the less stable of the two interacting Aap molecules fully unfolds, while the other remains in a folded conformation.

Short-ranged adhesion preceding unfolding events was frequently observed for WT–WT pairs, even after mAbs_A_ treatment, but was lacking in the absence of zinc or between WT and *∆aap* cells, indicating they involve B repeats between the two interacting cells. Recent experiments revealed that B-repeats feature a monomer–dimer–tetramer reversible equilibrium in the presence of zinc, resulting in the formation of functional amyloid fibers within the biofilm (23, 39). Therefore, a possible explanation is that the short ranged events involve the separation of multimeric Aap assemblies formed at the cell–cell interface. Hence, when applying force between two interacting cells, multimer assemblies at the interface would rupture first until the cells remain in contact only through homophilic bonds.

A unique finding of our study is that Aap also mediates cell–cell adhesion via heterophilic interaction between its lectin A subdomain and carbohydrate ligands. The A domain is known to promote *S.epidermidis* adhesion to host surfaces (24, 25, 35, 40), and the lectin subdomain was recently shown to mediate cell attachment to corneocytes by interacting with glycoproteins, and possibly glycolipids, on the skin cells (25). Yet, the A domain has never been shown to play a role in bacterial intercellular adhesion. We have discovered that the lectin domain specifically binds to *N*-acetyl-d-glucosamine through moderate forces that are typical of lectin-sugar interactions. *N*-acetyl-d-glucosamine is a monosaccharide known to be a component of the peptidoglycan polymer in the bacterial cell wall that is exposed on the surface of staphylococcal cells. Teichoic acids and CWA can also be glycosylated with *N*-acetyl-d-glucosamine on the staphylococcal cell surface (41, 42). We predict that the lectin-domain-mediated heterophilic interaction might thus exist in a broader scenario, that is, between any cells exposing glucosamine on their surface.

In summary, this study demonstrates the multifunctional roles of Aap in intercellular adhesion. We propose a model whereby fast lectin binding occurs when two cells contact each other, then with time conformational and/or orientational changes of the rod-like B repeats would allow strong homophilic bonds to form. Understanding the mechanisms involved in cell–cell interactions at play during biofilm formation may provide insights for the development of novel antiadhesive therapies specifically targeting those processes.

## Methods

### Bacterial strains and growth conditions

*Staphylococcus epidermidis* CSF 41498 (wild-type strain) and *S.epidermidis* CSF 41498 *∆aap* mutant were used in this study. Bacteria were cultured on brain heart infusion (BHI) agar plate for 24 h. One colony was inoculated in 10 mL BHI broth at 37°C overnight under shaking at 180 rpm. Bacteria were harvested by centrifugation three times for 5 min at 2000 × *g* and rinsed with Tris-buffered Saline (TBS, Tris 50 mM, NaCl 150 mM, pH 7.4), and resuspended in TBS buffer (instead of classical PBS to avoid zinc phosphate precipitates).

### Single-cell force spectroscopy

The bacterial suspensions were diluted 100× in TBS for the AFM experiments. A drop of the suspension was deposited on the bottom of a plate, incubated for 10 min, rinsed with TBS, and then 3 mL of TBS were added. Because of the zinc dependence of the interaction, zinc at a final concentration of 1 mM (10 min incubation) was added in the medium after chelating the preexisting zinc of the buffer with EDTA 1 mM (10 min incubation) to avoid a background effect unless mentioned. Single-cell probes were obtained by attaching a single bacterium to a colloidal probe. Colloidal probes were obtained as described below. Briefly, a small droplet of UV-curable glue (NOA 63, Norland Edmund Optics) was spread on one side of a glass slide and a silica microsphere (6.1 mm of diameter, Bangs Laboratories) on the other side. Triangular tipless cantilevers (NP-O10, Bruker) were brought first into contact with the glue manually using a Nanowizard III or IV AFM (JPK Instrument, Berlin, Germany) and then moved to catch a single silica microsphere. After that, the colloidal probe was exposed to a UV lamp for 15 min to cure the glue. Finally, the colloidal probe was incubated in a 10 mM Tris–HCl buffer solution (pH 8.5) containing 4 mg mL^−1^ dopamine hydrochloride for an hour, and washed in the same buffer. The nominal spring constant of these cantilevers was around ∼0.08 N m^−1^, as determined by the thermal noise method. Single-cell probe preparation: 50 μL of diluted bacteria suspension in TBS was deposited on a Petri dish and allowed to adhere for 15 min at room temperature. The Petri dish was then carefully washed twice to remove nonadhering cells, after which 3 mL of TBS buffer was added to perform AFM experiments. The colloidal probe was brought into contact with a single isolated bacterium to catch it via electrostatic interaction with polydopamine and then moved on top of another cell to probe its surface. Force–distance curves were recorded at a loading force of 0.25 nN, surface delay time of 0 s, constant approach and retraction speed of 1 µm s^−1^, and a Z closed loop at room temperature. For LR experiments, different retraction speeds were used: 1, 3, and 10 µm s^−1^, respectively. Images of 16 × 16 pixels or 32 × 32 pixels were recorded on areas of 500 nm × 500 nm on the bacterial surface. For each condition, experiments were repeated for at least four different pairs, from at least three independent bacterial cultures.

### Cross and blocking experiments

For cross experiments, a drop of each WT and mutant suspension (prepared as described above) were deposited on a Petri dish on two different and separate areas of the surface. The sample was then cared as in classical single-cell experiments (see above). During the experiment, the colloidal probe was brought into contact with a single isolated WT bacterium to catch it. The cantilever was then retracted and moved to the “mutant area,” where the bacterial probe was brought into contact with a single isolated mutant bacterium. Force–distance curves were collected in force volume mode using a constant approach and retraction speed of 1 µm s^−1^, a ramp length of 1 µm, an applied force of 250 pN, and a Z closed loop. 16 × 16 pixel maps were recorded on 500 nm × 500 nm areas of the mutant surface at room temperature. For sugar blocking experiments, after the recording of a map in the normal conditions, the cantilever was withdrawn and d-mannose or *N*-acetyl-d-glucosamine solution (Sigma) was injected in the medium, at a final concentration of 1 mM. Fifteen minutes after, the injection the same pair of cells (WT and WT, or WT and *∆aap* mutant) was used to record a map in the new conditions using the same parameters as before. For antibody blocking experiments, mAbs raised against the Aap region A (53–608) (mAbs_A_) were generated as described by Köhler and Milstein (33) with minor modifications and produced essentially as previously reported (34). Thirty microliters of mAbs_A_ (1 mg mL^−1^) was injected to reach a final concentration of 10 µg mL^−1^. After 15 min, a cell pair (WT and WT, or WT and *∆aap* mutant) was used to record a map using the same parameters as before.

### Data analysis

Force–distance curve data were analyzed using the data processing software from JPK (Berlin, Germany). Adhesion peaks were fitted using the WLC model. Unfolding forces, peak-to-peak distances, rupture forces, and rupture lengths were further analyzed and plotted with Origin Software. For dynamic force spectroscopy data, E and G5 unfolding forces were fitted with the BE model. Sample sizes and replicates are reported in the figure captions as well as in the main text. Statistical analyses were performed using GraphPad Prism version 8.0.2. Differences in data distributions between groups were analyzed using two-way Mann–Whitney *U* tests. For all experiments, a *P* value <0.05 was considered as significant.

## Supplementary Material

pgac278_Supplemental_Files

## Data Availability

Data supporting the findings of this manuscript are included in the published article and the supplementary information file. Source data are provided with this paper.
