# Supplementary material for: The staphylococcal biofilm protein Aap mediates cell–cell adhesion through mechanically distinct homophilic and lectin interactions
Source: PNAS Nexus. 2022 Dec 2;1(5):pgac278. doi: 10.1093/pnasnexus/pgac278 (PMC9802226; doi:10.1093/pnasnexus/pgac278)
Supplement: pgac278_Supplemental_Files [file pgac278_supplemental_files.zip › PNASNEXUS-PNASNEXUS-2022-00916-T-s01.pdf]

SI Appendix

The staphylococcal biofilm protein Aap mediates cell-cell adhesion through mechanically distinct homophilic and lectin interactions

Can Wang[a,1], Constance Chantraine[a,1], Albertus Viljoen[a], Andrew B. Herr[b], Paul D. Fey[c], Alexander R. Horswill[d], and Marion Mathelié-Guinlet[a,2,3], Yves F. Dufrêne[a,3]

[a]Louvain Institute of Biomolecular Science and Technology, UCLouvain, Croix du Sud, 4-5, bte L7.07.07, B-1348 Louvain-la-Neuve, Belgium

[b]Divisions of Immunobiology and Infectious Diseases, Cincinnati Children's Hospital Medical Center, Cincinnati, OH 45229, USA

[c]Department of Pathology and Microbiology, University of Nebraska Medical Center, Omaha, Nebraska 68198, USA

[d]Department of Immunology and Microbiology, University of Colorado School of Medicine, Aurora, Colorado 80045, USA

[1]C.W. and C.C. contributed equally to this work.

[2]Current address: Institut de Chimie et Biologie des Membranes et des Nano-Objets, CNRS UMR 5248, University of Bordeaux, IPB, 33600 Pessac, France.

[3]To whom correspondence may be addressed. Email: yves.dufrene@uclouvain.be or marion.mathelie-guinlet@u-bordeaux.fr

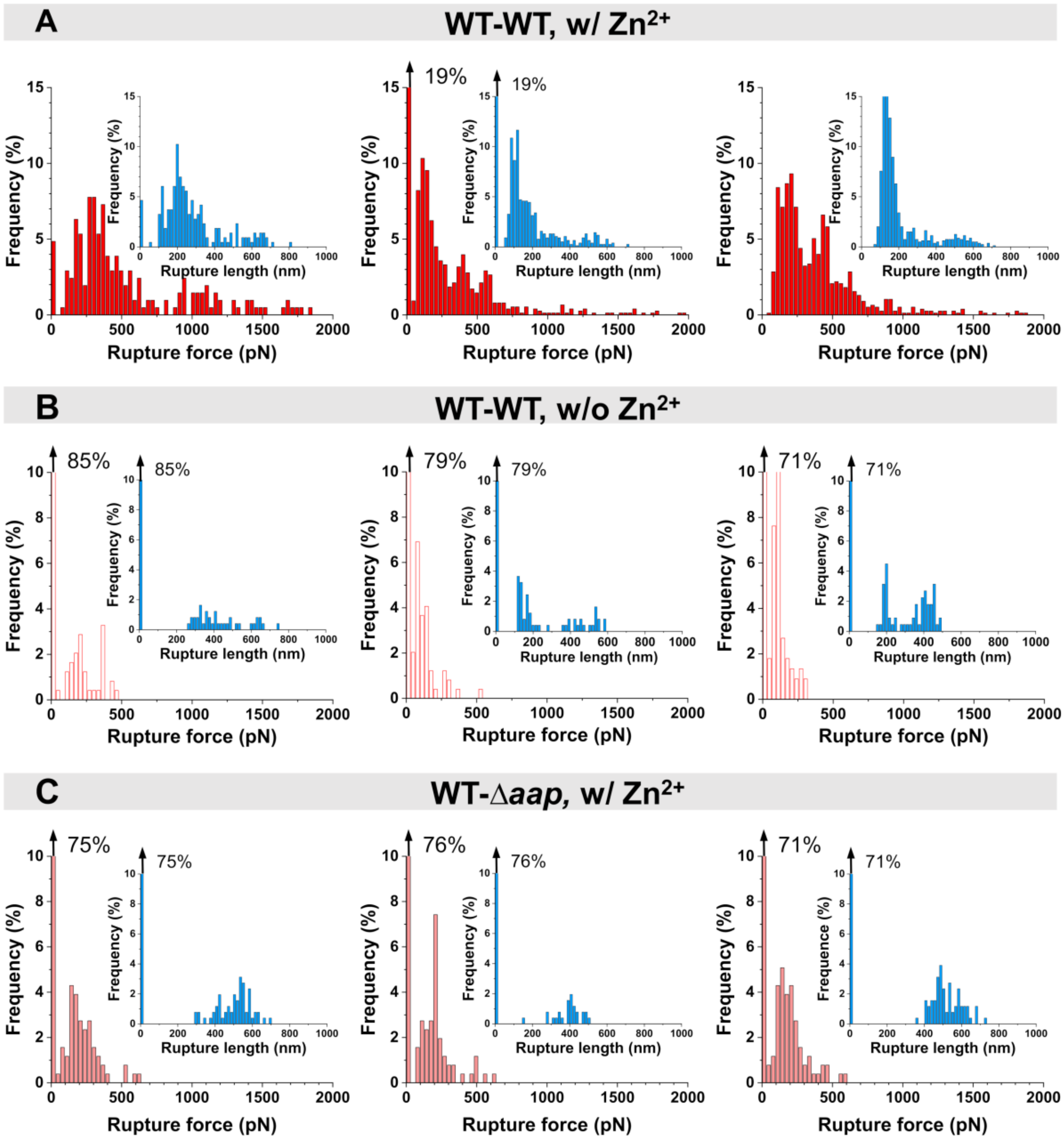

Fig. S1. Aap mediates *S. epidermidis* CSF41498 intercellular adhesion. Rupture force and rupture length (inset) histograms obtained by recording force-distance curves in TBS between *S. epidermidis* WT cell pairs with (**A**) or without (**B**) 1 mM Zn^{2+}. (**C**) Same data for WT-*Δaap* cell pairs in the presence of zinc (*n* = 256 curves for each cell pair). If present, the arrow at the top left of a histogram stands for the non-adhesive events.

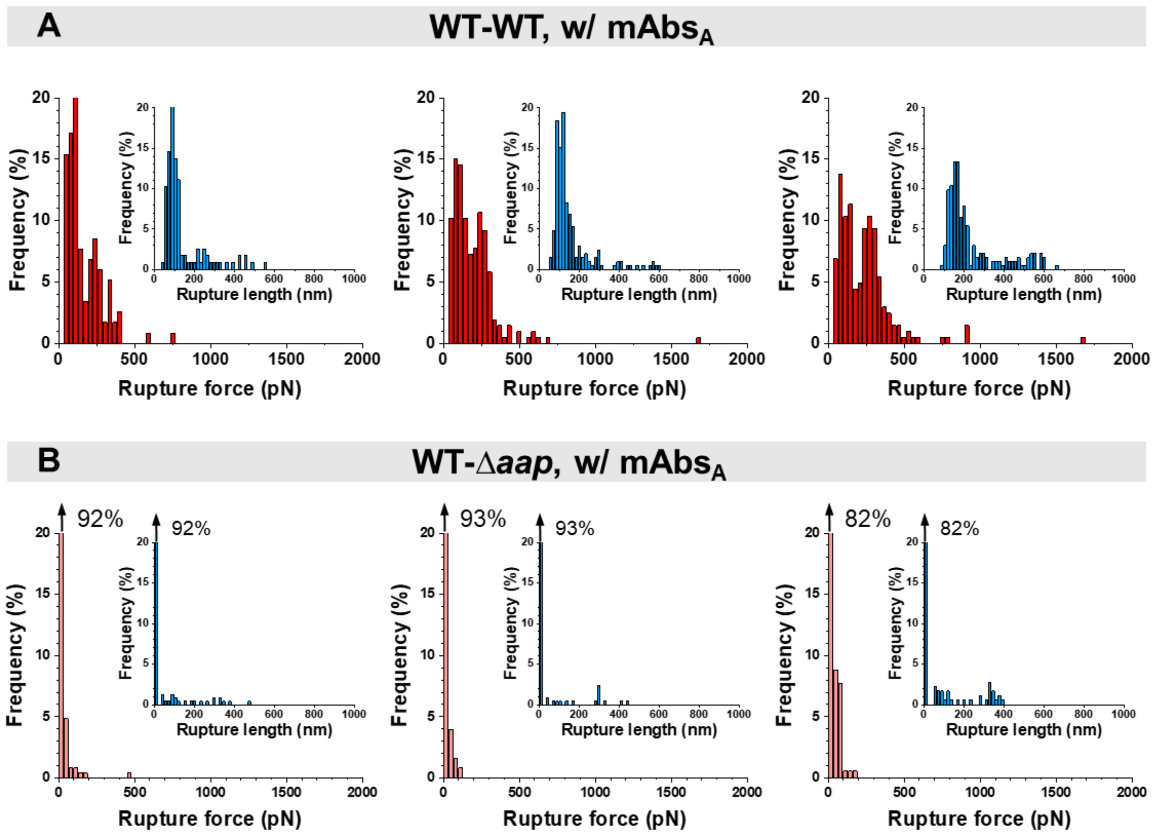

Fig. S2. The Aap A-domain engages in cell-cell interactions. Histograms of rupture force and rupture length (inset) for WT- *Δaap* pairs (**A**) and WT-WT pairs (**B**) after injection of 1 mM monoclonal antibodies directed against the A domain of Aap ($mAbs_A$). If present, the arrow at the top left of a histogram stands for the non-adhesive events.

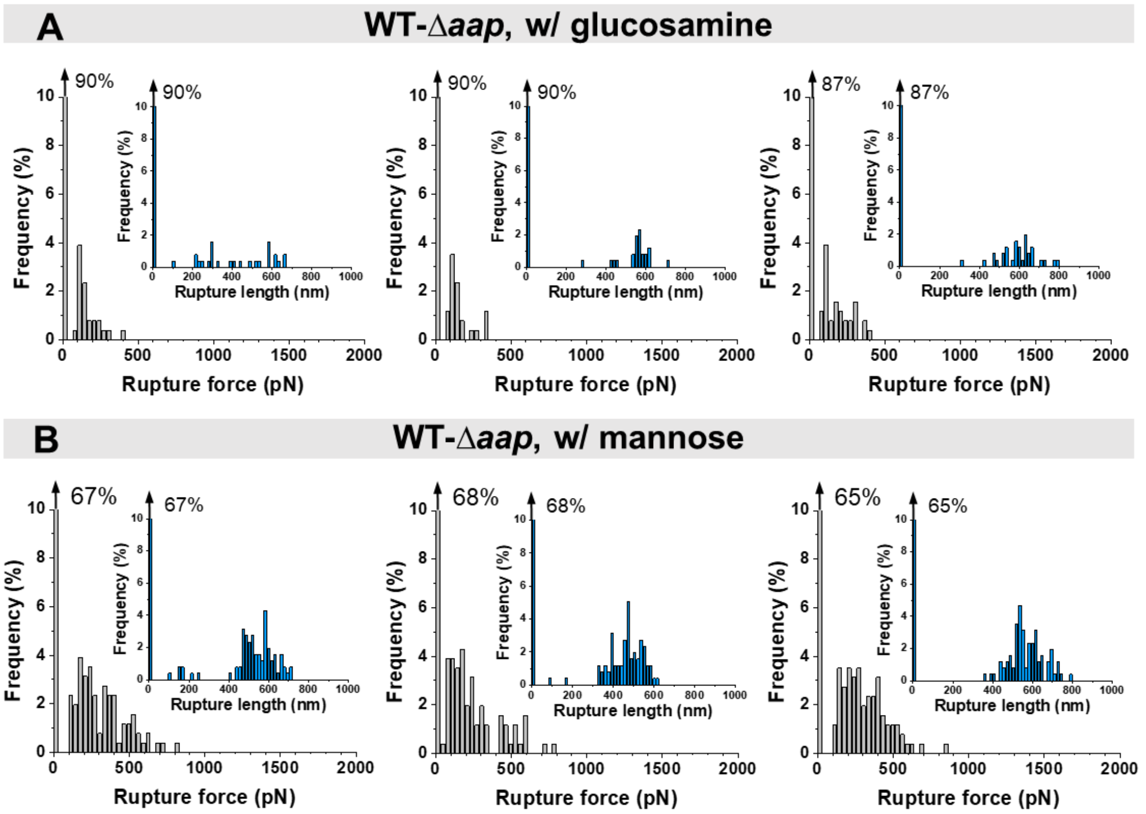

Fig. S3. Cell aggregation involves lectin-sugar binding. Histograms of rupture force and rupture length (inset) for WT-*Δaap* pairs after addition of 1 mM N-acetyl-D-glucosamine (**A**) or 1 mM mannose (**B**). The arrows at the top left of histograms stand for the non-adhesive events.

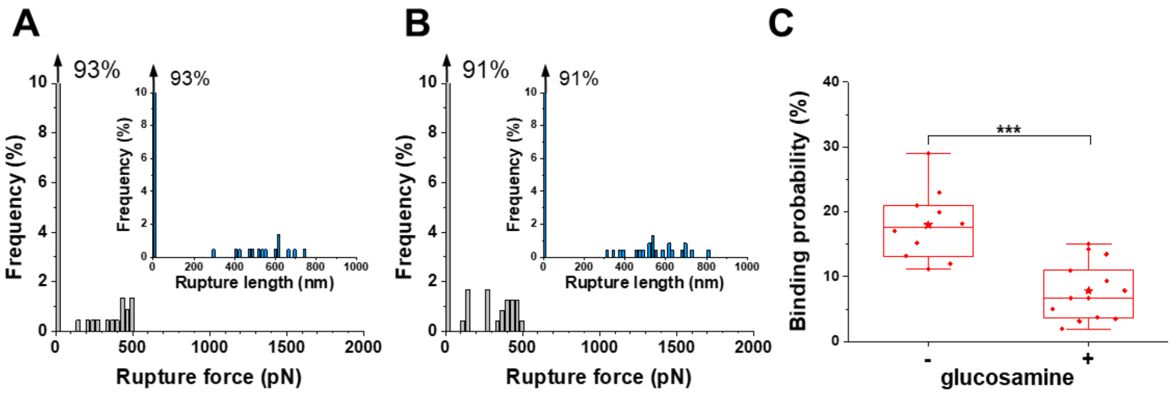

Fig. S4. Inhibition effect by N-acetyl-D-glucosamine on WT-WT interactions. (**A, B**) Rupture force and rupture length (inset) histograms for two representative WT-WT pairs after addition of 1 mM N-acetyl-D-glucosamine in absence of zinc. (**C**) Box plot comparing the binding probability between WT-WT cells, in the absence of zinc, before (n = 10 pairs) and after (n =

13 pairs) injection of 1 mM N-acetyl-D-glucosamine. Stars are the mean values, lines the medians, boxes the 25-75 % quartiles and whiskers the SD. ***, $P < 0.001$. The arrows at the top left of histograms stand for the non-adhesive events.
